# Long-Term Follow-Up of Multiple Autotransplantations Combined With Orthodontic Treatment After Traumatic Dental Injury: A Case Report

**DOI:** 10.1155/crid/7098948

**Published:** 2025-06-05

**Authors:** Johan Willem Booij, Barbara Disconzi, Marco Serafin, Alberto Caprioglio, Dick Barendregt

**Affiliations:** ^1^Private Practice, Gorinchem, the Netherlands; ^2^Department of Biomedical, Surgical, and Dental Sciences, University of Milan, Milan, Italy; ^3^Department of Biomedical Sciences for Health, University of Milan, Milan, Italy; ^4^Fondazione IRCCS Ca' Granda Ospedale Maggiore Policlinico, Milan, Italy; ^5^Proclin Rotterdam, Rotterdam, the Netherlands

**Keywords:** autotransplantation, case report, dental traumatology, multidisciplinary treatment, orthodontic treatment

## Abstract

In this case report, we present the treatment of an adult patient who had experienced dental trauma resulting in the loss of one tooth and damage to two others. The patient was referred to our clinic 4 years after the accident, where a comprehensive examination revealed external root resorption and loss of supporting tissue. To address the patient's concerns, we developed an interdisciplinary treatment plan, which included orthodontic treatment to level the curve of Spee, reduce the deep bite, and extrude the affected teeth. After that, we extracted the damaged Teeth 2.1 and 2.2 due to their poor prognosis and replaced them with autotransplantation of Teeth 1.4 and 3.1. Before the procedure, both teeth underwent endodontic treatment, and 6 weeks later, they were built up to aesthetically and functionally replace the lost teeth. Additionally, postautotransplantation orthodontic treatment was conducted to close the donor site space and bring the autotransplanted teeth to an ideal position. The interdisciplinary approach and successful treatment of this case suggest that autotransplantation can be a valid option for restoring compromised teeth, especially in adult patients who require orthodontic treatment. In conclusion, autotransplantation offers multiple benefits, including preserving the natural dentition, avoiding the use of dental implants or prostheses, and achieving excellent aesthetic and functional results. Furthermore, this case underscores the importance of individualized treatment planning and collaboration among dental specialists to achieve optimal outcomes for patients.

## 1. Introduction

Traumatic dental injuries are a significant public health problem because of their frequency, impact on economic productivity, and quality of life [1]. Epidemiologic studies have found that approximately one-third of children and one-fourth of adolescents and adults have suffered dental trauma; most of these injuries involve the maxillary central incisors [2]. Literature includes protocols, methods, and documentation for the clinical evaluation and treatment of traumatic dental injuries [3]. The options for these injuries depend on several factors related to the trauma, the patient, and the operator.

Transplantation of a tooth from one site into the receiving alveolus of the same patient, known as autotransplantation, is an important treatment option for the replacement of a lost or hopeless single tooth and is an alternative to dental implants or conventional prosthetic rehabilitation [4]. Tooth autotransplantation can be divided into three types: conventional replantation, the one that is reported in this article, whose procedure consists of the transplantation of an unerupted or erupted tooth in the same patient, from the extraction site to another one or to a new surgically prepared socket; intra-alveolar transplantation, a procedure in which the remaining dental portion is moved to a more coronal position in the same socket in which the tooth was located originally (uprighting, rotating, and extruding) [4]; and intentional replantation, applied for the extraoral endodontic procedure of a tooth that is immediately repositioned inside its alveolus [5]. The three surgical techniques are interrelated because they manage teeth that cannot reliably be treated using conventional endodontic, periodontal, or restorative approaches. Additionally, these methods follow a similar therapeutic process involving careful tooth extraction, direct examination of the tooth and its root surfaces, and eventual reimplantation.

Predictability of tooth autotransplantation and its prognosis can be affected by preoperative, intraoperative, and postoperative conditions. Several authors have identified a series of prognostic factors for autotransplantation: the patient's age and gender, the developmental stage and the root anatomy of the donor tooth, the alveolar bone support in all dimensions at the recipient site, the use of an atraumatic surgical procedure, the method of stabilization of the teeth immediately after transplantation, and their postoperative care [6].

Autotransplantation of teeth has been attempted for several centuries. However, it has not gained acceptance until recently because of the relevant rate of surgical complications in the past due to the lack of knowledge [7]. Once the biological principles and proper clinical techniques underlying successful autotransplantation are understood, it can be considered a highly successful treatment modality [4, 7, 8]. The preservation of the periodontal ligament and root canal system, as well as proper selection of the tooth to be transplanted, are all critical factors in the success of the procedure. By carefully managing these biological principles, dental professionals can provide patients with a safe and effective alternative to traditional tooth replacement options.

In recent years, significant attention has been given to autotransplantation as a viable treatment modality, particularly regarding molars, yet clinical documentation on autotransplantation in anterior teeth remains limited [9–11]. This gap highlights an important clinical need, considering anterior dental autotransplantation can significantly impact aesthetics, functionality, and psychological well-being following dental trauma [12].

Here, we report the 5-year long-term successful outcome of multiple conventional tooth replantation in conjunction with orthodontic and restorative treatments to replace one lateral and one central incisor in an adult patient who sustained trauma to the anterior maxilla.

## 2. Case Presentation

This case report has been prepared following the CARE (CAse REport) guidelines to ensure accurate and transparent reporting of clinical case information.

A 22-year-old woman with a history of dental trauma was referred to the orthodontic clinic by her general dentist. At the age of 19.4 years before the consultation, she had an accident with her horse in which she suffered trauma to her upper teeth caused by the animal kick: Tooth 2.3 was lost and Teeth 2.1 and 2.2 were luxated, along with multiple fractures of the alveolar bone surrounding the upper anterior teeth. Also, the patient reported a hairline fracture to the frontal region that resulted from the secondary impact against the concrete floor. Remarkably, the mandibular, nasal, and zygomatic bones remained unaffected.

Teeth 2.1 and 2.2 were reimplanted and splinted. Subsequently, these teeth, together with Tooth 2.4, underwent conservative restorative treatment to compensate for residual spacing and periodontal defect. In addition to these physical injuries, the patient experienced significant psychological trauma related to this incident.

**Figure 1:**
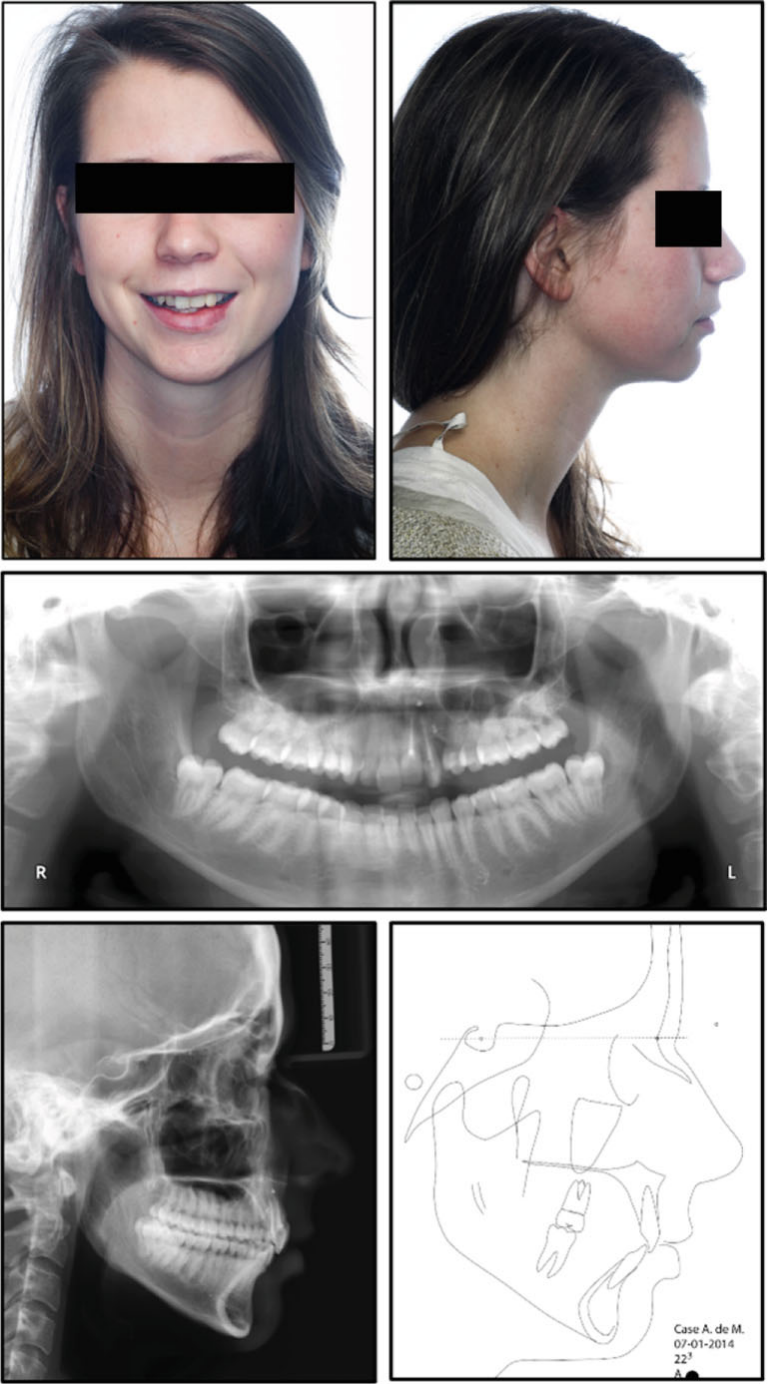
Facial analysis, panoramic and lateral radiographs, and cephalometry before treatment start.

The patient had a symmetrical face, good tooth exposure when smiling, a slight deviation of the midline to the left, a straight profile, and good lip closure (Figure 1).

**Figure 2:**
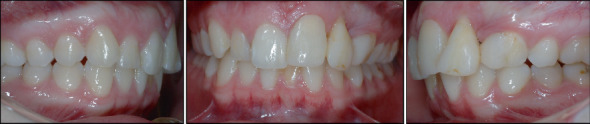
Intraoral photographs at treatment start.

The pretreatment panoramic radiograph showed external root resorption of Teeth 2.1 and 2.2, which had already undergone endodontic treatment after the accident, and the presence of radiopaque material apically and distally of Tooth 2.2. Dilaceration of Tooth 1.5, well-formed condyles, and the presence of Teeth 1.8, 2.8, 3.8, and 4.8 with impaction of Teeth 3.8 and 4.8 were also observed. Intraoral examination showed absence of Tooth 2.3 and loss of supporting periodontal tissue in Area 2.2, which was due to the accident (Figure 1). Occlusal relationships consisted of a Class II Division 2 malocclusion, with Class 2 molar relation on both sides, 3.5 mm overjet, 4 mm overbite, and 2.2 mm midline deviation (Figure 2).

Both the maxillary and mandibular arches were well formed, but the maxillary incisors were rotated, and the mandibular anterior teeth were crowded. The cephalometric radiograph showed a Class II Division 2 anomaly, good position of the lower incisors, good lip closure, a straight profile, and a normal nose (Figure 1). Finally, functional examination revealed no craniomandibular dysfunction symptoms and coincidence of centric relation with centric occlusion.

The primary objective was to establish a treatment plan that included replacement of Teeth 2.1 and 2.2, which required extraction due to a poor prognosis following dental trauma. The secondary objective was to achieve an esthetic result in the maxillary anterior region. The tertiary objective was to improve the occlusal relationship between the upper and lower arch to achieve a good functional occlusion.

The following treatment alternatives were considered and proposed to the patient:

− Guided bone regeneration (GBR) and dental implants to replace Teeth 2.1 and 2.2.
− Autotransplantation of Teeth 1.4 and 3.1 into the region of Teeth 2.1 and 2.2, followed by orthodontic treatment to close all gaps and achieve good functional occlusion. At the end of the orthodontic treatment, the autotransplanted teeth were to be restoratively and aesthetically reconstructed.

The patient and the interdisciplinary team opted for the second alternative because it was considered to offer the best long-term prognosis and only natural elements were used. Therefore, the patient underwent all the following treatments after the acceptance of the proposed interdisciplinary plan and provision of written informed consent.

**Figure 3:**
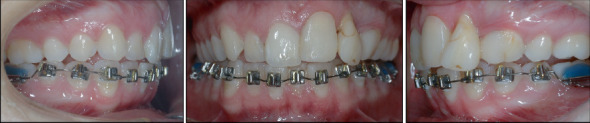
Lower arch bonding.

Treatment began with an upper bite plate used in combination with a Begg's fixed appliance in the lower arch to level the curve of Spee and reduce the deep bite (Figure 3).

All teeth were bonded, and a regular Australian 016” wire was placed as the first arch. The patient was instructed to always wear the bite plate. The irregular position of the lower anterior teeth was not corrected at this stage.

After 2 months, the upper bite plate no longer needed to be worn due to adequate bite opening. A fixed appliance was placed in the upper arch for slow extrusion of Tooth 2.1. This tooth was regularly reduced incisally to compensate for the extrusion. After 7 months, the situation was ready for the first transplantation.

**Figure 4:**
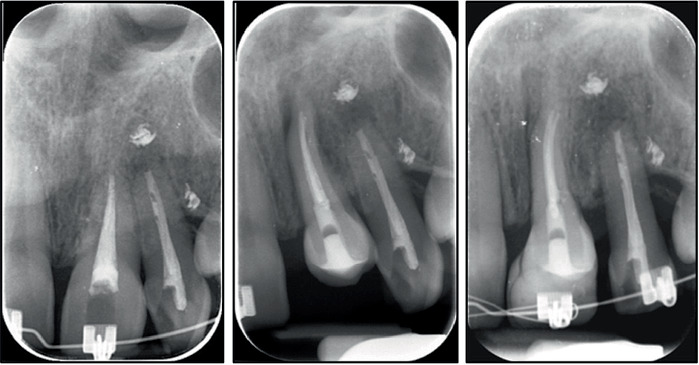
Radiographic evaluation of Tooth 2.1 during extrusion and Tooth 1.4 after its transplantation and bonding.

At 16 months, Tooth 1.4 was endodontically treated and transplanted to Tooth 2.1's location 1 month later (Figure 4).

**Figure 5:**
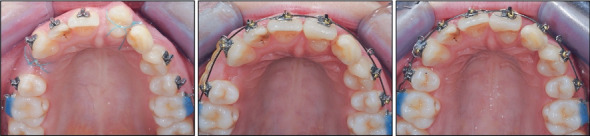
Intraoral details of autotransplantation of Tooth 1.4 to Tooth 2.1's site, its reconstruction, and extrusion of Tooth 2.2.

After suture removal at 1 week and check-up after 3 weeks for periodontal healing, a composite abutment was fabricated after 6 weeks, and a bracket was provided directly on the transplanted tooth by her general dentist (Figure 5).

An orthodontic wire was placed to functionally load the periodontal ligament of the new Tooth 2.1.

**Figure 6:**
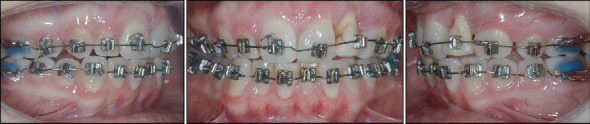
Extrusion of Tooth 2.2 for vertical development of the alveolar bone.

Slow extrusion of Tooth 2.2 with a 016” special plus Australian wire was then started (Figure 6).

This tooth was regularly reduced incisally to compensate for the extrusion too. At the same time, Tooth 1.3 was distalized to improve canine relation and close the extraction diastema.

**Figure 7:**
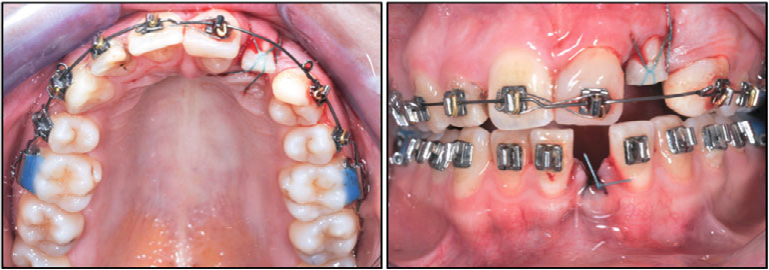
Intraoral details of autotransplantation of Tooth 3.1 to Tooth 2.2's site.

At 29 months, Tooth 3.1 received an endodontic treatment, and 1 month later, it was transplanted to Area 2.2 (Figure 7).

**Figure 8:**
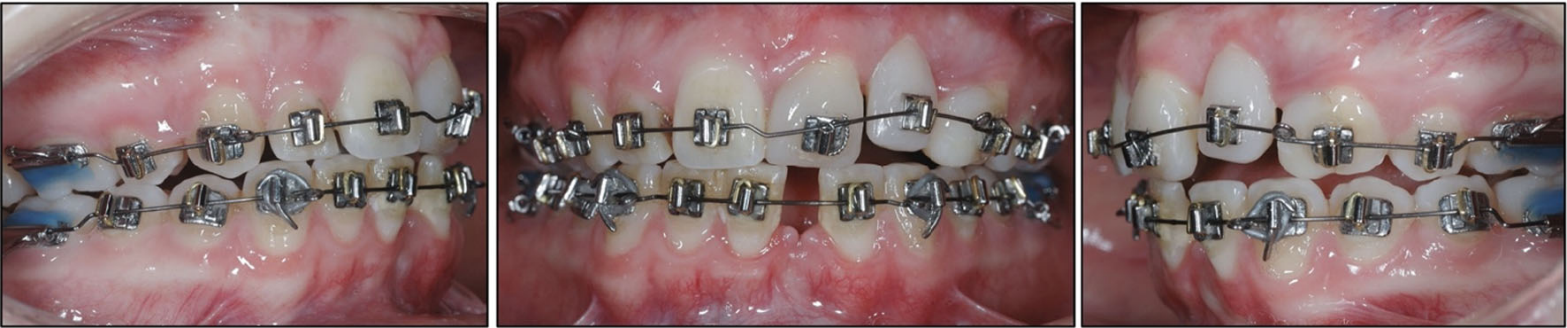
Reconstruction and bonding of the transplanted Tooth 3.1.

Subsequently, sutures were removed after 1 week, followed by a periodontal healing assessment at 3 weeks. At 6 weeks, the transplant was built up with composite by her general dentist, followed by direct bracket placement (Figure 8) and functional loading to stimulate the periodontal ligament.

**Figure 9:**
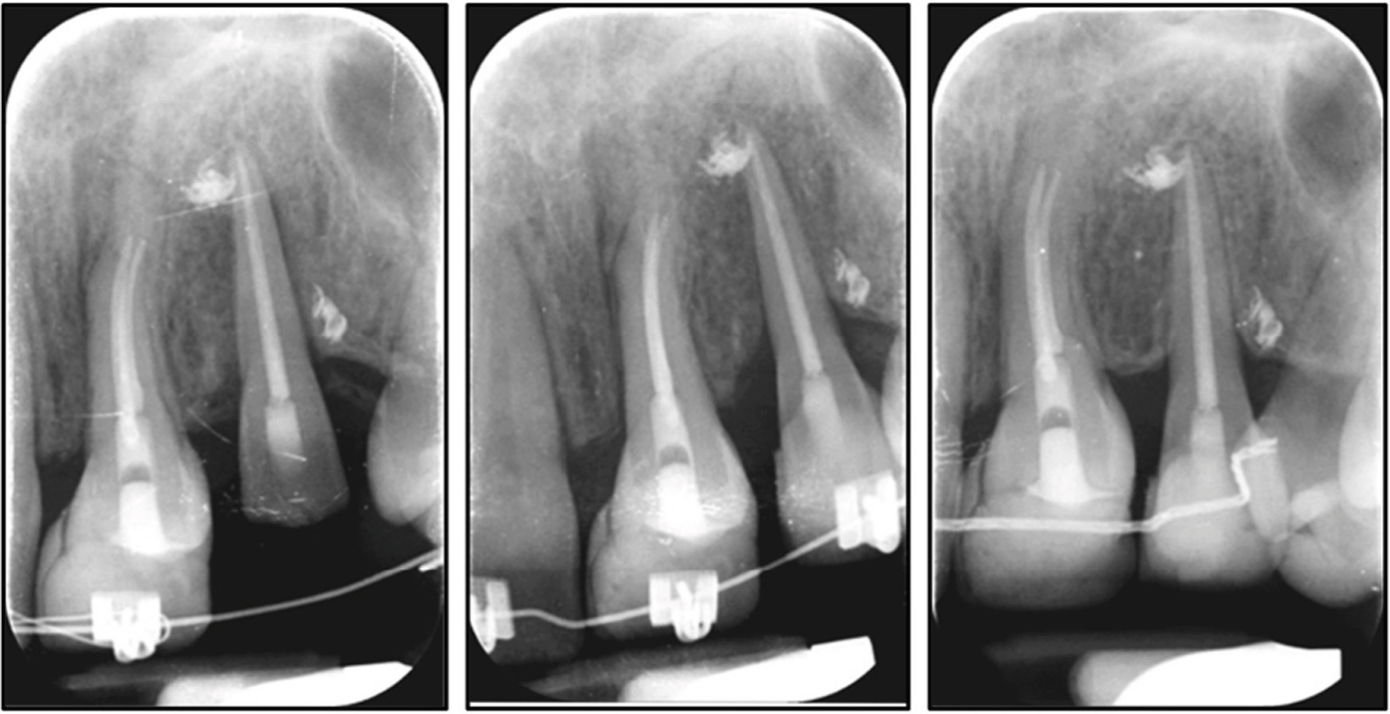
Radiographic evaluation of Tooth 3.1 during extrusion and after its transplantation and bonding.

Tooth extrusion was also followed radiographically to check periodically the vertical development of alveolar bone (Figure 9).

The process of closing the extraction space in the lower front was initiated. Detailing and finishing took some time, and it was finally decided to start retention and place two fixed retention wires.

**Figure 10:**
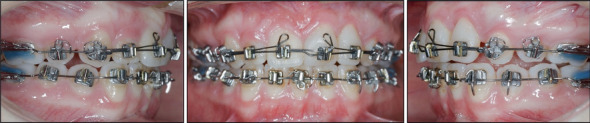
Detailing and finishing before final direct restorations.

At 62 months, a retreatment with a fixed appliance in the upper arch was initiated to close diastemas after new restorations and to torque and upright Tooth 2.1 (Figure 10).

At 74 months, the fixed appliance in the upper jaw was removed, and a fixed retainer in combination with Essix was applied.

**Figure 11:**
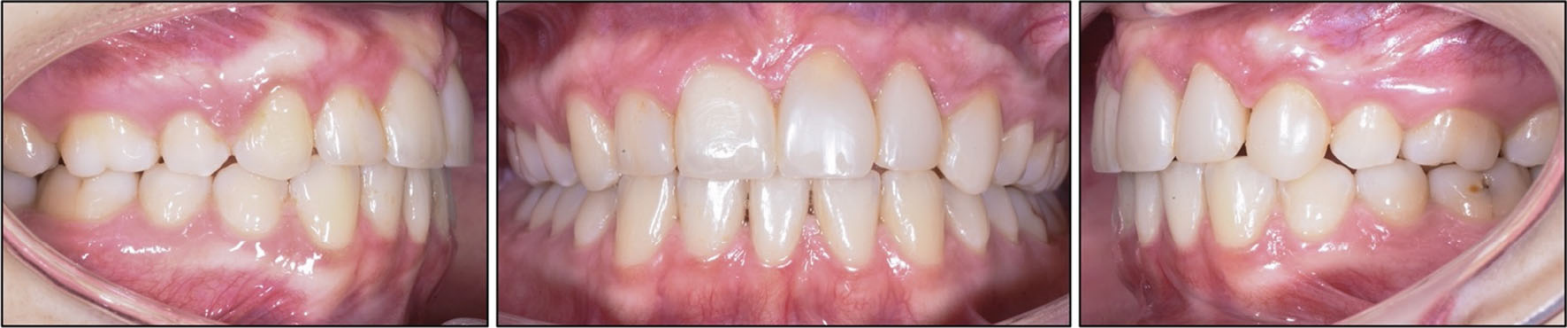
Intraoral radiographs at treatment end.

**Figure 12:**
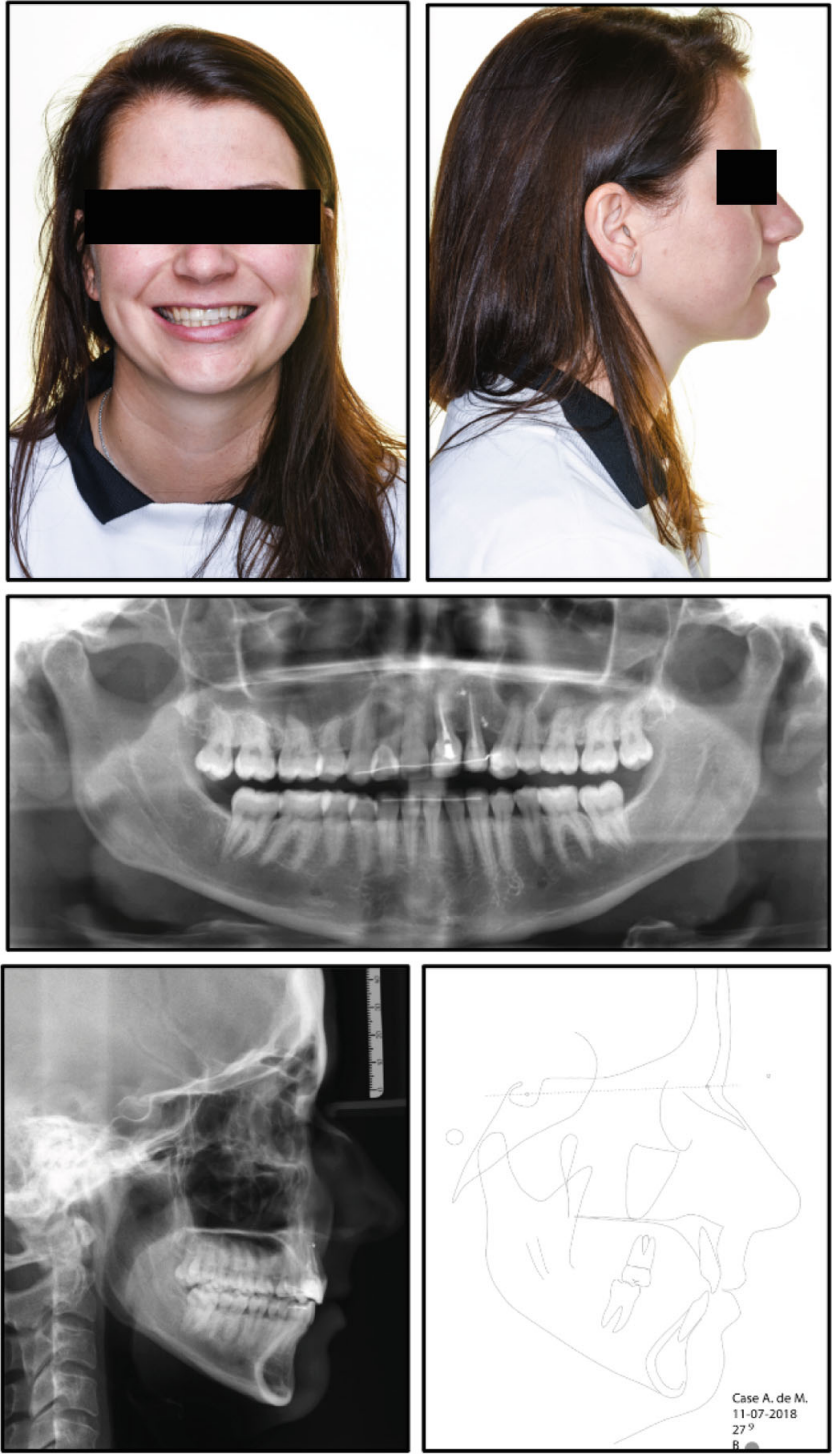
Facial analysis, panoramic and lateral radiographs, and cephalometry after treatment end.

The retention was limited to the placement of two bonded retainers to achieve optimal settling in the lateral regions. The patient was advised to keep the bonded retainers and to wear the upper Essix retainer at night. During the retention phase, Tooth 1.8, which had overerupted due to lack of occlusion, had to be removed. Finally, intraoral records (Figure 11) as well as facial photographs, a new panoramic and lateral radiograph, and cephalometric analysis (Figure 12) were recorded.

**Figure 13:**
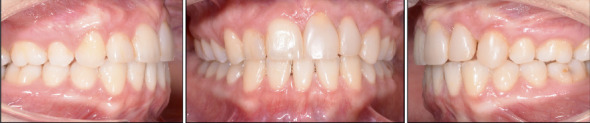
Five-year follow-up intraoral photographs.

**Figure 14:**
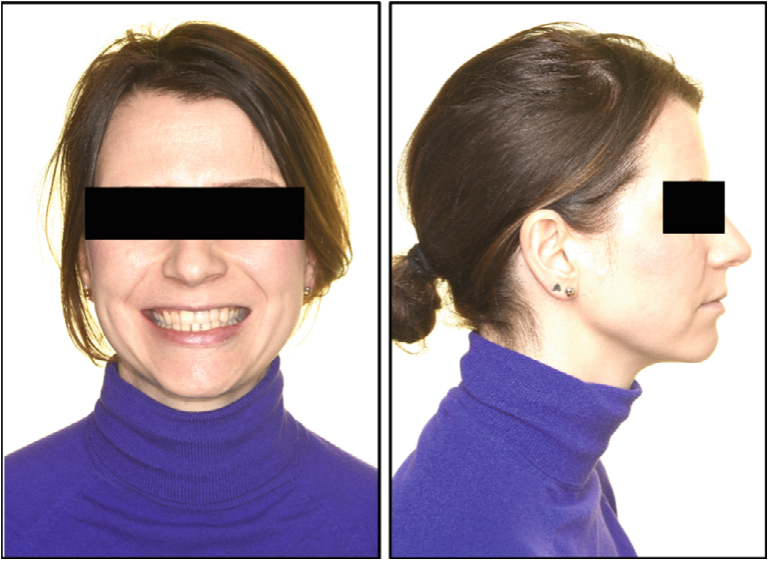
Five-year follow-up extraoral photographs.

Finally, long-term follow-up included extraoral and intraoral photographs taken at 5 years from the treatment end (Figures 13 and 14), showing the good integration between the transplanted teeth and the smile.

**Figure 15:**
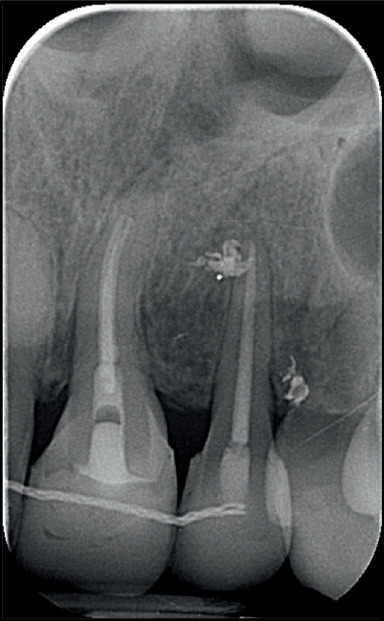
Radiographic follow-up after 8 and 7 years since the autotransplantation of Teeth 2.1 and 2.2, respectively.

In addition, 8 and 7 years of X-ray follow-up were recorded since the autotransplantation of Teeth 2.1 and 2.2, respectively (Figure 15).

The total treatment duration was 74 months. At the end, the patient was pleased with the results, considering the interdisciplinary approach had resulted in esthetically pleasing replacement of the compromised left central and lateral incisors, using only natural elements. The patient had a harmonious face, with relaxed soft tissues, good lip closure, and upper incisors in the midline. Despite the patient's age, superimposition showed that some growth of the mandible had occurred and there was no bite opening effect from the treatment. The correction of malocclusion was achieved through dentoalveolar changes that led to Class II molar and Class I premolar occlusion, with incisal and canine guidance.

Maxillary changes were represented by the torque of upper incisors, slight extrusion of upper first molars, while mandibular changes consisted of slight retrusion of lower incisors. Posttreatment radiographic evaluation of the autotransplanted teeth demonstrated all signs of success, which included no considerable root resorption and improvement of bone level in Areas 2.1, 2.2, and 2.3. However, the first composite restorations on Areas 2.1, 2.2, and 2.3 were not satisfactory. The difference between crown length left and right was disturbing, and optimal oral hygiene palatally of the upper front was difficult. One of the restorative dentists of the clinic managed to convince the patient to undergo a short follow-up orthodontic treatment to torque Tooth 2.1 and extrude Tooth 2.2 a little more for an optimal starting point to redo the composite rebuild on Areas 2.1, 2.2, and 2.3. After 6 months, the final composite restorations were placed. The trauma and injury to her teeth had a significant impact on the woman's psychological well-being.

## 3. Discussion

We described a case report in which autotransplantation, combined with orthodontic and restorative therapies, effectively replaced compromised central and lateral incisors in an adult female patient with a history of trauma to the anterior maxillary region.

One of the common indications for autotransplantation is the replacement of tooth lost due to trauma. Other main indications are congenitally missing teeth and developmental dental anomalies. Lastly, tooth lost to restorative or endodontic problems and periodontal or periapical inflammation can be replaced by the use of autotransplantation [6]. Autotransplantation represents a viable alternative therapeutic option to dental implants and fixed partial dentures.

The statement that autotransplantation represents a viable alternative to dental implants and fixed partial dentures indeed merits deeper exploration, particularly concerning contemporary regenerative procedures [13]. While implants often necessitate adjunct regenerative techniques, such as GBR, soft tissue augmentation, and the use of biomaterials, to achieve optimal aesthetic and functional outcomes, autotransplantation inherently utilizes the patient's own biological resources, thus minimizing or eliminating the need for these additional regenerative interventions [14]. In this case report, the interdisciplinary orthodontic management provided sufficient natural alveolar bone remodeling and soft tissue adaptation, highlighting a notable advantage of autotransplantation.

Compared to other prosthetic techniques, autotransplantation offers numerous advantages: use of an autogenous donor, preservation of aesthetics, and a functional replacement against reduced costs [15]. The main advantage of the autologous donor is the presence of a viable periodontal ligament. This brings several improvements, such as rebuilding of soft and hard tissues, preservation of proprioception, adaptive eruption, and the possibility of moving the tooth according to orthodontic treatment [16]. Damage to the periodontal ligament due to necrosis can lead to complications such as ankylosis, external root resorption, loss of periodontal attachment, and mobility [17, 18]. The main limitation of this technique is the availability of a donor tooth.

In dentistry, there is a long experience with transplanting developing teeth in the Scandinavian countries, while transplantation of mature teeth was mainly performed in Japan. Donor teeth with incomplete root formation have been preferred for transplantation because the vitality of the pulp can be preserved through the process of revascularization [19]. However, the selection of only such teeth significantly limits the indications for autotransplantation, since root formation has already been completed in all teeth in most adults.

Even autotransplantation of mature teeth may be considered a beneficial treatment if the donor tooth is endodontically treated [8, 18, 20]. Favorable success rates have been reported where the timing of the endodontic treatment and splinting modalities, tooth morphology, and systemic antibiotics are factors of influence of the outcome [8, 20, 21]. Rare failure rates, resorption rates, and ankylosis rates have been associated with autotransplantation of mature teeth [22].

In this case, the interdisciplinary team opted for autotransplantation to replace the compromised Teeth 2.1 and 2.2, even though root formation of the Donor Teeth 1.4 and 3.1 had been completed. The other relevant treatment alternative was dental implants and GBR. Since the patient was still at an age that significant skeletal adaptation in the anterior was to be expected, leading to infraocclusion of the implant-supported crowns, the best option was chosen [23].

Transplants and implants are two techniques with similar goals, so a comparison is inevitable [24]. Previous studies reported comparable survival rates of osseointegrated implants and autotransplanted teeth. For instance, previous studies indicated that autotransplanted teeth exhibited a survival rate of 90% during a follow-up ranging from 17 to 41 years, whereas single-tooth implants had a survival rate of 94.5% over a period of 5 years [25]. However, success rates differ greatly. For transplants, success after 10 years is, on average, over 90% with adults in the anterior up to 87.5% [21, 22]. For dental implants, after 5 years, a success of 61.3% is achieved [26].

There are some limitations that restrict the widespread use of implant rehabilitations, such as patient age, need for orthodontic treatment, and disposable income, making the autotransplanted option appear more favorable [16]. Autotransplantation, on the other hand, requires the presence of a donor tooth in the oral cavity and the fulfillment of certain conditions.

According to the current state of the art, autotransplantation in combination with orthodontic treatment should be considered as the first treatment alternative for a missing or hopeless tooth when a suitable donor tooth is available. Single implant rehabilitation should only be considered if the prognosis of conservative or surgical alternatives is unfavorable [27]. In fact, this case report underscores a fundamental yet frequently overlooked principle in dentistry: prioritizing biology over mechanical solutions [28]. Although single-tooth implants remain a common choice due to their predictable outcomes, clinicians sometimes neglect the intrinsic biological advantages offered by conservative surgical techniques such as autotransplantation.

## 4. Conclusions

The results of our case report suggest that tooth autotransplantation is an excellent biologic treatment alternative for the replacement of compromised teeth, especially in adults undergoing orthodontic treatment.

This technique has significant advantages over implants, and moreover, from the patient's point of view, the dentition can be preserved with a natural tooth instead of a mechanical prosthesis.

## Data Availability

The data that support the findings of this study are available on request from the corresponding author. The data are not publicly available due to privacy.
